# Optimising Nutrition and Hydration in Care Homes—Getting It Right in Person Rather than in Policy

**DOI:** 10.3390/geriatrics4010001

**Published:** 2018-12-20

**Authors:** Alison I. C. Donaldson, Alexandra M. Johnstone, Phyo K. Myint

**Affiliations:** 1 Ageing, Clinical and Experimental Research Group (ACER), School of Medicine, Medical Sciences and Nutrition, University of Aberdeen, Scotland AB25 2ZD, UK; alisondonaldson@nhs.net; 2 The Rowett Institute of Nutrition and Health, University of Aberdeen, Scotland AB25 2ZD, UK; alex.johnstone@abdn.ac.uk

**Keywords:** nutrition, dehydration, care homes, ageing, frailty

## Abstract

The scoping review by Bunn et al. identifies an important, but often invisible, challenge of malnutrition and specifically sub-optimal hydration and nutrition in the care home environment. Those requiring residential care are generally the frailest members of society, and likely to be affected by the anorexia of ageing: a multifactorial process whereby older people fail to adequately regulate food and nutrient intake resulting in unintentional weight loss. Adequate training of all healthcare professionals to recognise the risk of malnutrition at an early stage is fundamentally important, and the window of opportunity for intervention may be at a much earlier stage than admission to the care home. The specific needs of older adults must be considered in planning interventions with regard to the effects of ageing on physiology, digestion, and absorption of nutrients. Most importantly, we must offer person-centred care which offers residents an element of personal choice in whether or not they wish nutritional intervention, and any intervention offered must have the effect of improving quality of life rather than numbers on a scale.

The scoping review by Bunn et al. identifies an important, but often invisible, challenge of malnutrition and specifically sub-optimal hydration and nutrition in the care home environment [1]. Their insightful overview of the 79 documents pertaining to policies for nutrition and hydration in UK care homes, confirms that a plethora of information is available, but why is the UK prevalence of dehydration (20%) and malnutrition (34%) among older people living in care homes still seemingly high [2,3]? 

The global prevalence of malnutrition in the care home environment was estimated by means of pooled datasets across five continents by Kaiser et al in 2010, with a figure of 14.4% for males and 13.5% for females [4]. They also estimated the prevalence of those residents at risk of malnutrition to be 52.4% and 53.7% for males and females, respectively [4]. So how do we explain this discrepancy between global and UK estimates? Does our care home population represent a frailer cohort compared to other nations? Differences in institutionalisation rates between different countries have been shown, but nonetheless it is important that we carefully consider the means by which we can improve hydration and nutrition for this vulnerable group [5].

Those requiring residential care are generally the frailest members of society, and research has shown that community prevalence of frailty is higher in those with anorexia of ageing (20.3%) compared to those without (8.4%), therefore making it likely that those requiring residential care due to frailty are also affected by poor appetite [6]. The anorexia of ageing is a multifactorial process whereby older people fail to adequately regulate food and nutrient intake resulting in unintentional weight loss [7]. Factors influencing the progression of anorexia of ageing include altered chemosensory functions, impaired psychological function, changes in hormones regulating appetite and environmental changes [8,9]. The anorexia of ageing is also linked to other pathological conditions such as depression, dementia, somatic diseases, medications and iatrogenic interventions [10]. Moreover, aside from a means to satisfy hunger, eating represents a form of behaviour, so changing social circumstances, such as by a move to residential care, can impact on the reward factor of eating [11]. Currently there are no available management strategies to prevent or reverse the anorexia of ageing. Bunn et al highlight that changes in subjective motivation to eat have historically not been audited, although more recent work increasingly includes resident and carer perspectives [1].

The Global Leadership Initiative on Malnutrition recently recommended that screening for risk of malnutrition should be undertaken for anyone coming into contact with healthcare professionals [12]. However, this depends on healthcare professionals being adequately trained to identify malnutrition or those at risk, and to either manage this independently or refer patients on to a colleague who can. If we purely consider doctors, then the majority of junior American physicians (>75%) felt inadequately trained to counsel a patient on diet [13]. Moreover, a study of European medical schools suggested that nutritional education was a requirement in only 68.8% of institutions surveyed, with an average total of 24 hours for nutrition-based teaching [14]. Adequate training of all healthcare professionals to recognise risk of malnutrition at an early stage is fundamentally important, and should be a focus for continuous professional development for those working with older adults. It could be argued that identifying malnutrition at the point of admission to the care home setting is too late, and that the window of opportunity for intervention is at a much earlier stage.

Most UK care homes screen for malnutrition at the point of admission (>90%), and the majority utilise the Malnutrition Universal Screening Tool (MUST) (96%), but there is no currently validated tool for assessing dehydration risk in older adults [15,16]. Anecdotally, there have been concerns with possible user errors in MUST score calculation, particularly with comprehending the concept of percentage weight loss and identifying those that should score points for ‘acute illness’, potentially resulting in some care home residents in need of nutritional intervention being missed. However, perhaps a greater concern is whether identification of a care home resident at high risk of, or in a state of, malnutrition translates into adequate action. The four ‘nutrition week’ surveys by the British Association for Parenteral and Enteral Nutrition between 2007 and 2011 demonstrated considerable intra-individual variation in weight change between admission and the time of the survey, with nutritional status being identified as the key explanatory variable [15]. The survey demonstrated that those identified as ‘malnourished’ were most likely to be underweight on admission to a care home and to lose further weight during their residency; while the non-malnourished actually gained weight [15]. Evidently our current strategy is not working, so what can we change?

The specific needs of older adults must be considered in any food or beverages offered, particularly with regard to the effects of ageing on physiology, digestion and absorption of nutrients [10,17]. Knowledge of nutrition for healthy ageing is growing, and this should be used to tailor meal solutions in a ‘food first approach’. As an example, higher protein intakes (≥1.2 g/Kg/day) have been shown to decrease the risk of frailty, but we also now appreciate that the timing (pulse feeding rather than bolus feeding) and quality of protein supplementation is very important [10].

Solving the significant challenge of sub-optimal hydration and nutrition in the care home environment undoubtedly requires a multi-disciplinary approach from those at the fore-front of care and catering to those at the fore-front of nutritional research for healthy ageing. We must also not ignore the technology we have available to enable us to be innovators, not intervenors; a recent Dyson Award winner was motivated after watching his Grandma’s struggle with dementia to design hydrating ‘jelly drops’ which can deliver the equivalent of a cup of water in seven easy to eat ‘drops’ (https://www.jamesdysonaward.org/2018/project/jelly-drops/). 

Fundamentally, regardless of policy and guidelines, we must ask each individual resident what it is they want. We must offer person-centred care, which offers residents an element of personal choice in whether or not they wish nutritional intervention, and any intervention offered must have the effect of improving quality of life rather than numbers on a scale.
